# Spinal myoclonus following a peripheral nerve injury: a case report

**DOI:** 10.1186/1749-7221-3-18

**Published:** 2008-08-06

**Authors:** Feray Karaali Savrun, Derya Uluduz, Gokhan Erkol, Meral E Kiziltan

**Affiliations:** 1 Department of Neurology, Istanbul University Cerrahpasa Medical Faculty, Istanbul, Turkey

## Abstract

Spinal myoclonus is a rare disorder characterized by myoclonic movements in muscles that originate from several segments of the spinal cord and usually associated with laminectomy, spinal cord injury, post-operative, lumbosacral radiculopathy, spinal extradural block, myelopathy due to demyelination, cervical spondylosis and many other diseases. On rare occasions, it can originate from the peripheral nerve lesions and be mistaken for peripheral myoclonus. Careful history taking and electrophysiological evaluation is important in differential diagnosis.

The aim of this report is to evaluate the clinical and electrophysiological characteristics and treatment results of a case with spinal myoclonus following a peripheral nerve injury without any structural lesion.

## Background

Myoclonus is defined as a sudden muscular contraction that usually indicates disease of the central nervous system and may be cortical, subcortical, or spinal in origin [1]. Spinal myoclonus is a rare disorder characterized by myoclonic movements in muscles that originate from several segments of the spinal cord. Though structural lesions are usually found in spinal myoclonus, the pathophysiology remains speculative. But there is evidence that various possible mechanisms can be involved: loss of inhibitory function of local dorsal horn interneurons, abnormal hyperactivity of local anterior horn neurons, aberrant local axons re-excitations and loss of inhibition from suprasegmentar descending pathways [2].

This report describes a case with spinal myoclonus following a peripheral nerve injury. Clinical, electrophysiological characteristics and treatment results were discussed.

## Case presentation

A 33-year-old female was admitted to Neurology Department with a complaint of weakness, hypoesthesia, paresis and painless constant involuntary muscle spasms of the left upper extremity. Her complaints started 4 months ago, after she fell upon her left arm. At that time there appeared a collection and oedema on the left arm elbow joint. In a month, she experienced weakness, sensory deficits and minimal muscle spasms in the left ulnar nerve innervation area. Cervical magnetic resonance imaging (MRI) was normal. Electromyographic evaluation (EMG) revealed a conduction delay and/or a conduction block with a neurogenic involvement displaying partial denervation in muscles innervated by ulnar nerve. Collection was evacuated by decompression surgery and ulnar nerve was released. After the operation weakness and sensory deficits did not improve. Involuntary movements in the left ulnar nerve innervated muscles, than increased and spread to the the whole arm. She was referred to our clinic. Her family history was unremarkable. She was not on any medication, she did not smoke or drink alcohol. Neurological examination revealed spontaneous synchronized, involuntary myoclonic jerks in the proximal part of the left upper extremity during action and at rest (see Additional file 1).

Myoclonus seen in the agonist and antagonist muscles were persisting during sleep as her parents noted. It was provoked by movements that belonged to the affected muscle groups but there was no response to tactile stimulus. Minimal muscle weakness and sensory deficit in the biceps, triceps and brachioradialis muscles were noted.

Routine biochemical laboratory investigations were within normal limits. Secondary causes of myoclonus such as infectious disease (HIV, VDRL, HSV, hepatitis B and C, syphilis) were excluded. Blood calcium, copper, seruloplasmin levels, hepatic and renal function tests, thyroid hormone levels, sedimentation rates, cerebrospinal fluid findings and routine EEG and cranial MRI scanning were normal. Computerized tomography (CT) of the left arm, performed due to the trauma of left upper extremity, revealed a fissure, 1 cm above the humero-radial joint at the level of the lateral epichondylus. MRI of the forearm revealed a partial rupture in the collateral ligament that achive the stabilization of the wrist, a strain in the distal part of the triceps muscle and articular effusion.

**Figure 1:**
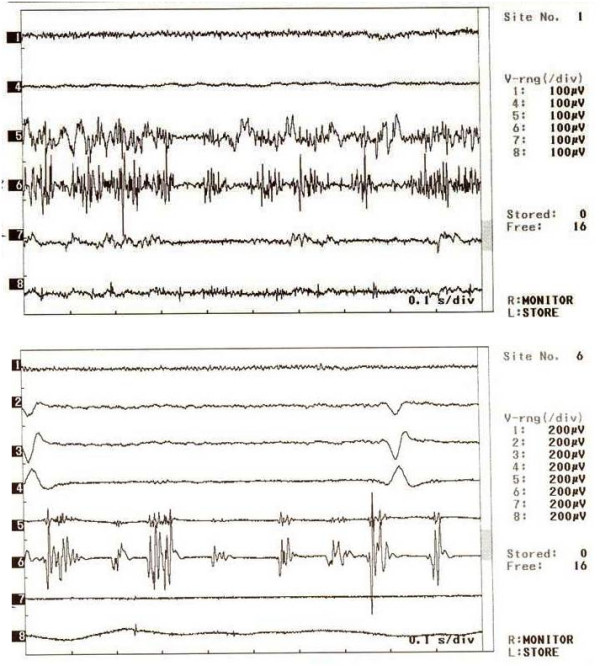
**EMG recordings with surface electrodes**. EMG channesl: 1-M. Orbicularis oris 2-M. Trapezius 3-M. Rhomboideus 4-M. Pectoralis 5-M. Biceps Brachii 6-M,. Triceps 7-Forearm flexor muscles 8-Forearm extansor muscles.

Needle EMG findings, motor and sensory nerve conduction studies of the upper extremity muscles were in normal limits. Somatosensorýal evoked potentials (SEP) were normal. The surface EMG showed rhythmic, irregular, 1–3 Hz in frequency discharges in motor units of muscles expanding from the fifth to the eighth cervical region of the left upper extremity in a segmented fashion (Figure 1). Agonist and antagonist muscle contractions and discharges were synchronized. The myoclonic activity started synchronously in the whole segment and there was no startle response in supraorbital, median, ulnar nerve electrical or auditory stimulation, which suggested that it was not stimulus-sensitive.

As a result of clinical, laboratory, radiological and electrophysiological evaluations, the patient was diagnosed as having a non-proprioceptive spinal myoclonus. Various drugs were used (Carbamazepine 800 mg/day, Na valproate 1000 mg/day, Piracetam 4.8 g/day, Clonazepam 6 mg/day) but none of them were effective. Since there was no response to medical treatment, botulinum toxin type A (Botox ^®^) was applied to the left extremity triceps and biceps muscles. After a week of botulinum toxin injection, a temporary improvement was noted but it was not considered to be satisfactory.

## Discussion

The label of spinal segmental myoclonus was appropriate if there is pathology in the spinal cord and the movements exist according to those segments, In our patient, both clinical and electromyographic findings pointed to the C5 to C8 segments as the site of segmental spinal myoclonus. The collection was evacuated and decompression was performed at the beginning, since there was ulnar nerve compression in the electrophysiological evaluation, but her sypmtoms did not subside. Cervical MRI taken after the trauma was normal. The findings were widespread and not limited to the ulnar nerve tract as expected. These movements were started following a trauma, suggesting that the disease might be triggered by peripheral nerve damage. In clinical and electrophysiological evaluations it was shown that the pathology progressed to the upper segments; above the area of the peripheral nerve. Propriospinal myoclonus affects multiple neighbouring segments. But, in our case, the movement was observed synchronously in the whole segment. Spinal myoclonus may be stimulus-sensitive as well but we did not observe any involvement such as a startle induced by a peripheral nerve or supraorbital stimulus; therefore, we concluded that the pathology was not a stimulus-sensitive type.

The diagnosis of psychogenic myoclonus was considered but a psychiatry consultation was completely normal. Furthermore, myoclonus continued during sleep and occurred synchronously in agonist and antagonist muscles.

Spinal myoclonus has been associated with laminectomy, remote effect of cancer, spinal cord injury, post-operative pseudomeningocele, laparotomy, thoracic sympathectomy, poliomyelitis, herpes myelitis, lumbosacral radiculopathy, spinal extradural block, myelopathy due to demyelination, electrical injury, acquired immunodeficiency syndrome, and cervical spondylosis [3]. In rare occasions, spinal myoclonus can be observed after the peripheral nerve lesions. Peripheral nerve lesion as a cause of spinal myoclonus is still the subject of debate.

There is evidence that various pathological mechanisms could be involved: e.g. loss of inhibitory function of local dorsal horn inter-neurons, abnormal hyperactivity of local anterior horn neurons, aberrant local axons re-excitations and loss of inhibition from supra-segmentar descending pathways [2].

The following findings support the reasons why the present case considered to be spinal myoclonus and not a peripheral one; the complaints started after a peripheral trauma and persisted, although decompression surgery was performed and even increased. It did not affect only the ulnar nerve tract, as in peripheral myoclonus, but involved the upper segments also and was widespread, had rhythmic and synchronous presentation, continued during sleep and was not stimulus-sensitive.

Clonazepam is the treatment of choice. Besides this Carbamazepine, Diazepam and Levatiracetam were tried in a few cases. In our patient, various medical treatments were applied (Clonazepam 6 mg/day, Carbamazepine 800 mg/day, Na valproate 1000 mg/day, Piracetam 4.8 g/day) but no response was observed. There are suggestions that botulinum toxin type A could be beneficial in cases resistant to medical treatment [4]. In our case, botulinum toxin was injected locally but it was not effective.

## Conclusion

In conclusion; spinal myoclonus can originate from the peripheral nerve lesion and be mistaken for peripheral myoclonus. While the underlying lesion is usually treatable and reversible in peripheral myoclonus, spinal myoclonus usually persists though various treatments. Careful history taking and electrophysiological evaluation is important in differential diagnosis.

## Competing interests

The authors declare that they have no competing interests.

## Authors' contributions

FK Carried out the electromyographical studies, participated in the conception and design of the manuscript as well as performed electromyographical examinations and material support. DU Carried out the clinical examinations, participated in the conception and design, acquisition of the data, and editted the revision of the manuscript. GE Carried out the clinical examinations and participated in conception and design of the data. MK Carried out the electrophysiological evaluations and participated as a supervisior. All authors read and approved the final manuscript.

## Consent

Written informed consent was obtained from the patient for publication of this case report and accompanying images. A copy of the written consent is available for review by the Editor in Chief of this journal.

## Supplementary Material

Additional file 1 Movie representing myoclonus. This movie shows the spinal myoclonus following a peripheral nerve injury. Click here for file
